# Exploratory Single-Nucleus RNA Sequencing Suggests Glial-Specific NPY Upregulation and Cell-Type-Specific Metabolic Alterations in Temporal Lobe Epilepsy

**DOI:** 10.3390/biology15080627

**Published:** 2026-04-16

**Authors:** Chao Jiang, Yan Zhao, Yaning Ding, Shanshan Wu, Le Su, Chenyang Bai, Jian Wang, Chuang Guo, Zhiqiang Cui

**Affiliations:** 1 Key Laboratory of Bioresource Research and Development of Liaoning Province, College of Life and Health Sciences, Institute of Neuroscience, Northeastern University, No. 195, Chuangxin Road, Hunnan District, Shenyang 110169, China; jiangchao103@163.com (C.J.); 19847421130@163.com (Y.Z.);; 2 Department of Neurosurgery, Chinese People’s Liberation Army of General Hospital, 28, Fuxing Road, Haidian District, Beijing 100853, China; 3 Graduate School, School of Clinical Medicine, North China University of Science and Technology, Tangshan 063000, China; 4 Medical School of Chinese People’s Liberation Army, Beijing 100853, China

**Keywords:** temporal lobe epilepsy, single-nucleus RNA sequencing, neuropeptide Y, microglia, oligodendrocytes, neuroinflammation, metabolic reprogramming

## Abstract

Temporal lobe epilepsy (TLE) is the most common focal epilepsy in adults, but cell-type-specific molecular alterations in the epileptic cortex remain incompletely characterized. We performed single-nucleus RNA sequencing on temporal cortex from three patients with drug-resistant TLE and two non-epileptic controls, retaining 66,932 nuclei. Seven major cell types were annotated. Neuropeptide Y (*NPY*) was significantly upregulated in microglia and oligodendrocytes under stringent criteria (|log_2_FC| > 1, adjusted *p* < 0.01), whereas changes in other cell types did not meet this threshold. Microglia showed enrichment of neuropeptide- and inflammatory-related pathways, together with reduced oxidative phosphorylation signatures. Oligodendrocytes showed altered lipid metabolism, together with reduced mitochondrial energy-related signatures. Inferred intercellular communication was globally reduced in the TLE samples. qPCR in an independent small set showed an upward trend of *NPY* expression, though not statistically significant. Given the limited cohort size, these results should be interpreted as exploratory. They provide a cell-type-resolved candidate framework for future mechanistic studies of glial-associated responses in human epilepsy.

## Simple Summary

Temporal lobe epilepsy is a common and often drug-resistant form of epilepsy. This study used single-nucleus RNA sequencing to examine all cell types in temporal cortex tissue from patients with drug-resistant temporal lobe epilepsy and non-epileptic controls. We found that neuropeptide Y, a molecule traditionally thought to come from neurons, was increased mainly in two types of support cells called glial cells—microglia and oligodendrocytes. These glial cells also showed distinct pathway changes related to metabolism and signaling. In addition, cell-to-cell communication appeared to be reduced in epilepsy samples. Because the study included only a small number of patients and lacked protein-level validation, these findings should be considered preliminary. Nonetheless, they provide a detailed cell-type-specific view of the epileptic brain and generate ideas for future research.

## 1. Introduction

Temporal lobe epilepsy (TLE) represents the most prevalent form of focal epilepsy, accounting for approximately 60% of all adult epilepsy cases worldwide [1,2]. Despite substantial advances in antiseizure medications over the past decades, nearly one-third of TLE patients develop drug resistance, requiring surgical intervention or alternative therapeutic approaches [1,2,3]. The disease burden extends far beyond seizure recurrence, as TLE is frequently accompanied by cognitive impairment, psychiatric comorbidities, including depression and anxiety, autonomic dysfunction, and increased mortality risk, including SUDEP (sudden unexpected death in epilepsy), profoundly affecting patients’ quality of life [4,5,6,7]. Understanding the molecular mechanisms underlying TLE pathogenesis remains a critical unmet need for developing disease-modifying therapies.

The pathogenesis of TLE involves complex, heterogeneous molecular alterations across diverse neural cell types. Traditional bulk RNA sequencing approaches, while providing valuable insights into global gene expression changes in TLE including apoptotic ceRNA networks in hippocampal sclerosis, are fundamentally limited in their ability to dissect cell-type-specific contributions within the heterogeneous neural tissue [8,9,10]. This limitation is particularly problematic given the recognized importance of neuron–glia interactions in epilepsy pathophysiology [11,12]. The emergence of single-cell and single-nucleus RNA sequencing (scRNA-seq/snRNA-seq) technologies has revolutionized our capacity to characterize transcriptomic heterogeneity, enabling the identification of cell-type-specific alterations and novel subpopulations, previously obscured in bulk analyses [9,13]. In this study, snRNA-seq was employed as a research tool to characterize cell-type-specific transcriptomic alterations in TLE rather than as a clinical diagnostic or biomarker assay. Our goal is to generate hypotheses for future mechanistic and therapeutic investigations [13].

Recent single-cell transcriptomic studies in TLE have begun to deconstruct this cellular complexity. Microglia adopt disease-specific activation states, including an iron-accumulating subtype (MG3), whose abundance correlates positively with seizure frequency [14]. Pro-inflammatory signaling (SPP1, TGF-β, and MHC-II) is enhanced across microglial populations, accompanied by peripherally derived T cells, extending neuroinflammation beyond innate immunity [8]. Astrocytes undergo profound reprogramming, with emergence of lipid-accumulated reactive astrocytes (LARAs) promoting disease progression [15] and a hybrid glial population co-expressing astrocyte and OPC markers, suggesting lineage plasticity [16]. Metabolic dysfunction emerges as a cross-cutting theme: mitochondrial impairment and altered lipid metabolism have been identified across cell types, with mitochondria-related genes such as *HSDL2* dysregulated in TLE astrocytes [2,15].

However, these studies have primarily focused on individual cell types in isolation or specific pathological features such as neuroinflammation or metabolism, leaving critical gaps in our understanding of shared molecular programs across glial lineages, cell-type-specific neuropeptide signaling beyond neurons, and alterations in intercellular communication networks in the epileptic human temporal cortex. Furthermore, whether glial cells express and regulate neuropeptides traditionally considered neuron-specific, such as neuropeptide Y (*NPY*), remains entirely unexplored.

Notably, *NPY*, a 36-amino acid peptide traditionally studied as a neuron-derived modulator of excitability and seizure threshold, has been implicated in epilepsy pathophysiology [17,18,19]. *NPY* exerts potent anticonvulsant effects through Y2- and Y5-receptor-mediated inhibition of glutamate release and neuronal hyperexcitability [17,18]. However, its cell-type-specific expression patterns in the epileptic human brain remain largely unexplored, particularly beyond established neuronal sources. Whether glial cells, which constitute the majority of brain cells and which are central to neuroinflammatory and metabolic responses, express and regulate *NPY* in epilepsy is unknown. Furthermore, comprehensive analyses integrating all major cell types, identifying shared dysregulated genes across populations, and mapping intercellular communication networks in the TLE temporal cortex remain limited.

Thus, critical knowledge gaps persist regarding the cell-type-specific molecular signatures across the epileptic temporal cortex, the reorganization of intercellular communication networks, and the lineage-specific contributions of metabolic dysfunction. A comprehensive, integrative analysis of how these processes are coordinated across distinct cell populations to shape the epileptic microenvironment is therefore warranted.

To address these gaps, we performed snRNA-seq on temporal cortex tissues from drug-resistant TLE patients and non-epileptic controls to systematically characterize cell-type-specific molecular signatures and disrupted cell–cell communication networks. Our specific aims were to: (1) identify and annotate major cell types in the human temporal cortex; (2) characterize cell-type-specific differentially expressed genes (DEGs) and enriched biological pathways, with focused analysis on microglia and oligodendrocytes; and (3) map alterations in intercellular communication networks between TLE and control samples. Through this integrative approach, we identified *NPY* as a candidate gene of interest showing significant upregulation in microglia and oligodendrocytes and subsequently assessed by qPCR in an independent sample set. By integrating these analyses, we provide a preliminary cell-type-resolved molecular landscape of TLE, suggesting cell-type-specific metabolic patterns that may inform future hypothesis-driven studies.

## 2. Materials and Methods

### 2.1. Study Participants and Tissue Samples

Temporal lobe cortex tissues were collected from three patients with drug-resistant TLE, who underwent curative surgical resection at the Department of Functional Neurosurgery, First Medical Center, Chinese PLA General Hospital, between 1 March 2023 and 31 October 2025. All TLE patients met the International League Against Epilepsy (ILAE) criteria for drug-resistant epilepsy and had hippocampal sclerosis confirmed by postoperative histopathological examination. The epileptogenic zone was localized through comprehensive preoperative evaluation, including long-term video electroencephalography (EEG), magnetic resonance imaging (MRI), and positron emission tomography-computed tomography (PET-CT). All TLE patients underwent preoperative video EEG monitoring for seizure focus localization. Antiepileptic medications were tapered or discontinued 3–7 days prior to surgery to facilitate seizure capture during monitoring, following standard clinical protocols [20,21]. This preoperative withdrawal period minimized acute drug effects on brain tissue gene expression.

Control temporal cortex tissues were obtained from two non-epileptic individuals undergoing neurosurgery at the same institution. One sample was derived from macroscopically normal temporal lobe tissue adjacent to a contused area but at least 2 cm from the primary injury site, resected during decompressive craniectomy for severe traumatic brain injury from a patient with no history of epilepsy. The other sample was adjacent normal temporal lobe tissue, more than 2 cm from the tumor margin, resected during benign meningioma surgery (WHO grade I). Both control individuals had no history of seizures or epilepsy and no epileptiform discharges on preoperative EEG. Postoperative pathological examination confirmed the absence of tumor infiltration in the collected control tissues. All control subjects were medication-naïve and did not receive any chronic or acute pharmacological treatment before surgery.

Detailed inclusion and exclusion criteria for both TLE patients and controls are provided in Supplementary Table S1. Clinical and pathological characteristics of all subjects, including age, sex, disease duration, MoCA scores, and specific pathological diagnoses, are summarized in Supplementary Table S2. All procedures were performed in accordance with the Declaration of Helsinki. This study was approved by the Ethics Committee of Chinese PLA General Hospital (approval number: S2022-038-01), and written informed consent was obtained from all subjects or their legal guardians.

### 2.2. Single-Nucleus Library Construction and Sequencing

Nuclei were isolated from frozen temporal cortex tissues following a demonstrated protocol (10x Genomics, CG000124). Briefly, the tissue was homogenized in ice-cold lysis buffer, incubated on ice, and filtered through a 40 μm strainer. After centrifugation, nuclei were purified through density gradient centrifugation to remove debris and myelin. The final nuclear pellet was resuspended in wash buffer to achieve a target concentration of 700–1200 nuclei/μL. Nuclei integrity was assessed through trypan blue staining.

Single-nucleus suspensions were loaded onto the Chromium Controller (10x Genomics, Pleasanton, CA, USA) for gel bead-in-emulsion (GEM) generation. snRNA-seq libraries were constructed using Chromium Single Cell 3′ Reagent Kit v3.1 (10x Genomics, Pleasanton, CA, USA) according to the manufacturer’s protocol. Libraries were sequenced on an Illumina NovaSeq 6000 platform (Illumina, San Diego, CA, USA) with paired-end 150 bp reads, targeting a minimum sequencing depth of 50,000 reads per nucleus.

### 2.3. Bioinformatics Analysis

#### 2.3.1. Data Preprocessing, Quality Control, and Clustering

Raw sequencing data were demultiplexed and aligned to the GRCh38 human reference genome using the Cell Ranger count pipeline (v9.0.1, 10x Genomics). Quality control was performed in Seurat v5.0.1: nuclei with <200 or >6000 detected genes, >25% mitochondrial UMI counts, or <500 total UMIs were removed. These thresholds were selected based on standard snRNA-seq practices to exclude empty droplets, doublets, and low-quality nuclei. Doublets were filtered using DoubletFinder v2.0.3. After Harmony-based batch correction, graph-based clustering (resolution = 0.6) and manual cell type annotation were performed, yielding 66,932 high-quality nuclei for downstream analyses. UMI count matrices were log-normalized, and the top 2000 highly variable genes were used for principal component analysis (PCA). The first 30 principal components were applied for graph-based clustering and non-linear dimensional reduction (t-SNE, UMAP).

#### 2.3.2. Cell Type Annotation

Initial cell type annotation was performed using scCATCH v2.0, followed by manual refinement based on canonical marker genes: neurons (*RBFOX3*, *SYT1*), microglia (*CX3CR1*), astrocytes (*AQP4*), oligodendrocytes (*MOBP*), oligodendrocyte progenitor cells (OPCs; *PDGFRA*), endothelial cells (*CLDN5*), and pericytes (*PDGFRB*). Feature plots and t-SNE plots were generated to verify marker specificity.

#### 2.3.3. Differential Expression Analysis

DEGs between TLE and control groups for each major cell type were identified using two complementary approaches. For within-cluster comparisons, the FindMarkers function in Seurat (Wilcoxon rank-sum test) was used. For group-level comparisons, we adopted a pseudo-bulk approach: UMI counts for each cell type were aggregated across all cells within the sample to generate pseudo-bulk expression profiles, followed by differential expression analysis using DESeq2. Pseudo-bulk results were prioritized for downstream interpretation to minimize single-cell-level noise and batch effects. Genes with an absolute average log_2_ fold change (|log_2_FC|) of >1 and an adjusted *p* value of < 0.01 (Benjamini–Hochberg correction) were considered statistically significant.

#### 2.3.4. Functional Enrichment and Cell–Cell Communication Analysis

Functional enrichment analysis of DEGs was performed using clusterProfiler v4.4.4. Gene Ontology (GO) terms and Kyoto Encyclopedia of Genes and Genomes (KEGG) pathways with *p* < 0.05 were considered significantly enriched. Given the stringent criteria applied for DEG selection and the exploratory nature of the pathway analysis, *p* values were not adjusted for multiple comparisons. To infer intercellular communication networks, CellChat v1.6.1 was applied to both TLE and control samples. Ligand–receptor pairs were identified based on the CellChat database, and communication probabilities were calculated. Signaling pathways with significantly different interaction strengths between conditions were identified and visualized.

#### 2.3.5. Identification and Prioritization of NPY as a Core Candidate

To identify key genes potentially driving TLE pathology, we performed a multi-dimensional screening strategy. First, we focused on genes differentially expressed in both microglia and oligodendrocytes based on pseudo-bulk DESeq2 results. Second, candidate genes were required to show enrichment in epilepsy-relevant pathways (e.g., neuroinflammation, synaptic signaling, and neuropeptide signaling) based on GO and KEGG analyses. Third, we examined ligand–receptor interactions involving microglia and oligodendrocytes from the CellChat analysis to identify genes potentially mediating intercellular crosstalk. Fourth, genes with previously reported clinical relevance in epilepsy were prioritized. Through this integrative approach, *NPY* emerged as a consistently dysregulated gene in both microglia and oligodendrocytes, ranking among the top five upregulated genes in these cell types, and was selected for experimental validation.

### 2.4. Quantitative Real-Time PCR (qPCR) Validation

Total RNA was extracted from frozen temporal cortex tissues using TRIzol reagent (Thermo Fisher Scientific, Waltham, MA, USA). cDNA was synthesized using the High-Capacity cDNA Reverse Transcription Kit (Applied Biosystems, Waltham, MA, USA). Primers for *NPY* and the endogenous reference gene, *HPRT1*, were custom-designed using Primer-BLAST (NCBI, https://blast.ncbi.nlm.nih.gov/Blast.cgi, accessed on 12 April 2026); the sequences are provided in Supplementary Table S3. qPCR was performed on an ABI QuantStudio 3 system using SYBR Green Master Mix (Thermo Fisher Scientific, Waltham, MA, USA). All reactions were run in triplicate. Relative expression levels were calculated using the 2^−ΔΔCt^ method. Statistical significance was assessed through two-tailed Mann–Whitney U test.

### 2.5. Statistical Analysis

Statistical analyses were performed using GraphPad Prism v9.0 and R (v4.2). For snRNA-seq analyses, the biological sample was treated as the statistical unit. UMI counts for each cell type were aggregated at the sample level to generate pseudo-bulk expression profiles, and differential expression was analyzed using DESeq2 (v1.38.0). Genes with |log_2_FC| > 1 and Benjamini–Hochberg-adjusted *p* < 0.01 were considered differentially expressed. Pathway enrichment and CellChat analyses were exploratory; nominal *p* < 0.05 was considered significant for descriptive purposes. For qPCR validation, given the very small sample size (*n* = 2–3 per group) and the lack of parametric assumptions, a two-tailed Mann–Whitney U test was used. The data are presented as median (IQR). No power calculation was performed; findings are exploratory.

## 3. Results

### 3.1. Single-Nucleus Data Quality Control and Cell-Type Annotation

After stringent quality control, batch correction, and cell type annotation, a total of 66,932 high-quality nuclei were retained from five samples (three TLE, two controls) for downstream analyses. Detailed sample-level quality control metrics are provided in Table 1.

**Figure 1:**
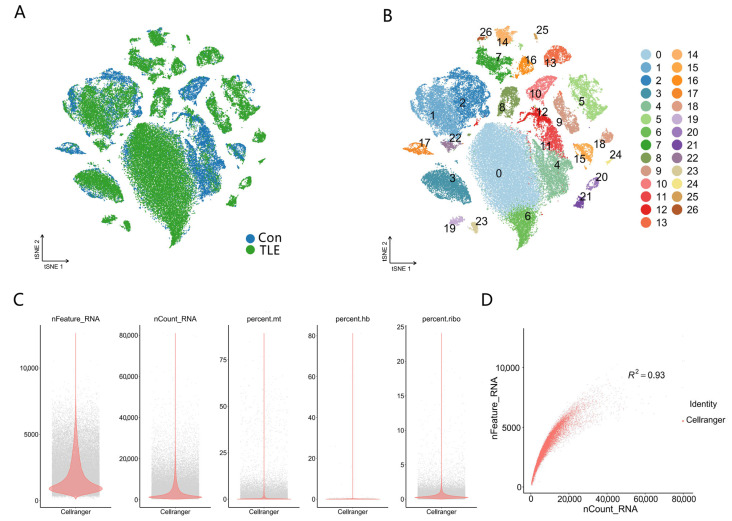
Data quality control and cell clustering results. (**A**) t-distributed stochastic neighbor embedding (t-SNE) visualization showing the separation of nuclei from TLE and control groups. (**B**) t-SNE visualization of graph-based clustering revealing 27 distinct cell clusters. (**C**) Violin plot of mitochondrial gene expression percentage across all cell clusters. (**D**) Scatter plot demonstrating the correlation between UMI counts and detected gene counts per nucleus.

**Figure 2:**
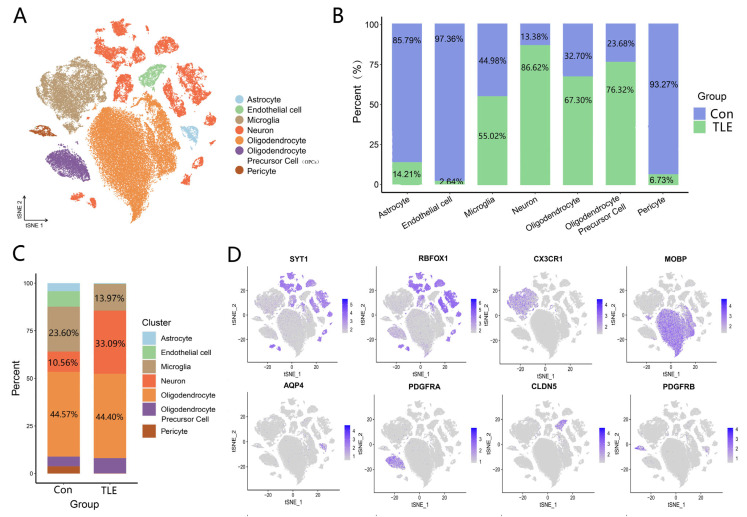
Cell type annotation and distribution in the human temporal cortex. (**A**) t-SNE visualization of seven major cell types annotated based on canonical marker genes. Each color represents a distinct cell type: neurons, astrocytes, microglia, oligodendrocytes, oligodendrocyte progenitor cells, endothelial cells, and pericytes. (**B**) Bar plot showing the proportion of each cell type in TLE and control groups. The *x*-axis indicates cell type, and the *y*-axis indicates percentage. (**C**) Stacked bar plot displaying the overall cellular composition of TLE and control groups, illustrating the relative abundance of each cell type within each condition. (**D**) Feature plots showing the expression of representative marker genes for each cell type: *SYT1*, *RBFOX1* (neurons), *AQP4* (astrocytes), *CX3CR1* (microglia), *MOBP* (oligodendrocytes), *PDGFRA* (OPCs), *CLDN5* (endothelial cells), and *PDGFRB* (pericytes). Color intensity represents expression level.

As shown in Figure 1A, t-distributed stochastic neighbor embedding (t-SNE) analysis revealed a clear separation between TLE and control nuclei. Unsupervised graph-based clustering followed by t-SNE visualization identified 27 distinct cell clusters, demonstrating the cellular heterogeneity of the human temporal cortex (Figure 1B). The percentage of mitochondrial gene expression was evenly distributed across clusters, with no cluster showing abnormally high mitochondrial content, confirming good cell viability (Figure 1C). Furthermore, a strong positive correlation between UMI counts and detected gene counts per nucleus indicated high-quality library preparation and sequencing depth (Figure 1D).

t-SNE visualization revealed distinct cell clusters that were annotated into seven major cell types based on canonical marker genes: neurons (*SYT1*^+^), microglia (*CX3CR1*^+^), astrocytes (*AQP4*^+^), oligodendrocytes (*MOBP*^+^), OPCs (*PDGFRA*^+^), endothelial cells (*CLDN5*^+^), and pericytes (*PDGFRB*^+^) (Figure 2A). The expression specificity of these markers was confirmed by feature plots (Figure 2D). A heatmap showing the top five most highly expressed genes in each cell type is provided in Supplementary Figure S2.

The distribution of cell types differed between TLE and control groups. TLE samples contained a higher proportion of neurons (33.09% vs. 10.56%) and lower proportions of microglia, astrocytes, endothelial cells, and pericytes compared to controls (Figure 2B,C; Table 2). Notably, the absolute number of neurons in TLE samples (14,923) was substantially higher than in controls (2305). This between-group difference should be considered when interpreting downstream comparisons.

### 3.2. Identification and Validation of NPY as a Core Candidate Gene

Differential expression analysis using stringent criteria (|log_2_FC| > 1, adjusted *p* < 0.01) revealed that *NPY* was significantly upregulated exclusively in microglia (log_2_FC = 4.35, adjusted *p* = 1.12 × 10^−11^) and oligodendrocytes (log_2_FC = 4.19, adjusted *p* = 5.32 × 10^−26^) in TLE compared to controls (Table 3). Although nominal upregulation was observed in neurons and OPCs, these changes did not reach the pre-specified significance threshold (Supplementary Table S4). No significant *NPY* expression change was detected in astrocytes, endothelial cells, or pericytes. For endothelial cells and pericytes, reliable differential expression analysis was further hindered by their extremely low nuclei counts in TLE samples (48 and 60 cells, respectively; Table 2).

Given the stringent significance criteria, we prioritized microglia and oligodendrocytes for downstream analysis. Microglia showed relatively comparable counts between groups (6300 vs. 5151), whereas oligodendrocytes remained imbalanced (20,025 vs. 9729) but substantially more interpretable than neurons, astrocytes, endothelial cells, or pericytes. These latter populations were excluded from detailed analysis due to insufficient cell numbers or severe between-group imbalance. Notably, among the analyzed populations, *NPY* upregulation was restricted to microglia and oligodendrocytes. Although significantly upregulated in these glial populations, *NPY* expression was confined to a subset of cells within each population rather than being broadly induced (Supplementary Figure S3).

**Figure 3:**
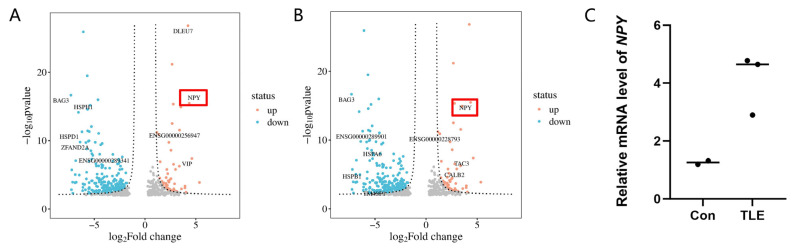
Glial *NPY* upregulation in TLE. (**A**,**B**) Volcano plots of DEGs in microglia (**A**) and oligodendrocytes (**B**). *NPY* is highlighted. (**C**) qPCR validation of *NPY* expression in bulk tissue.

To validate these transcriptomic findings at the mRNA level, we performed qPCR on bulk tissue RNA from an independent set of samples (control, *n* = 2; TLE, *n* = 3). The results showed an upward trend of *NPY* mRNA expression in TLE samples compared with controls, consistent with the glial-specific upregulation observed in the snRNA-seq data. However, the difference did not reach statistical significance (Mann–Whitney U test, exact *p* = 0.200, two-tailed; Figure 3C), likely due to the limited sample size and the dilution of cell-type-specific signals in bulk tissue.

### 3.3. Cell-Type-Specific Differentially Expressed Genes and Functional Enrichment

#### 3.3.1. Microglia

Microglia from the TLE patients displayed 14 upregulated and 36 downregulated DEGs. Combined GO and KEGG pathway enrichment analyses were performed. Supplementary Table S5 lists the top significantly enriched GO and KEGG terms, along with detailed gene compositions and *p* values (*p* < 0.05).

**Figure 4:**
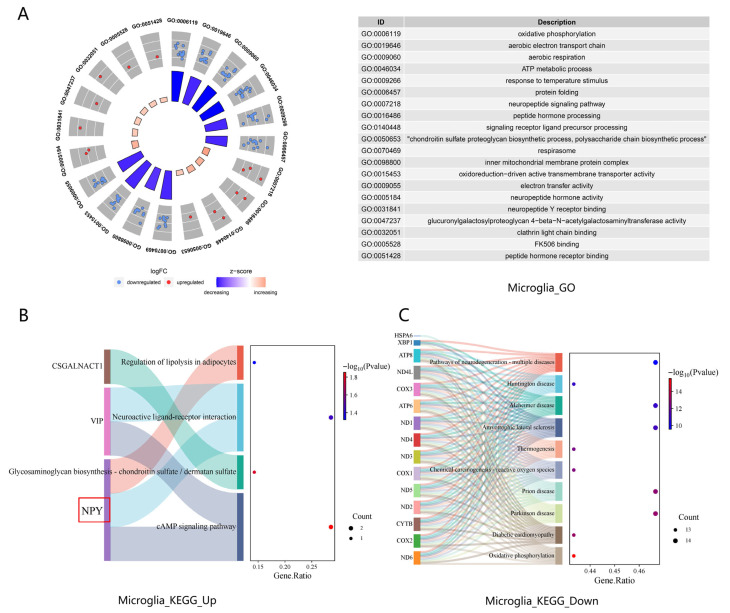
GO and KEGG enrichment analyses of DEGs in microglia between TLE and control groups. (**A**) Circular plot showing top enriched GO terms for upregulated (red) and downregulated (blue) genes (*p* < 0.05). (**B**) Sankey bubble plot of upregulated KEGG pathways (*p* < 0.05). (**C**) Sankey bubble plot of downregulated KEGG pathways (*p* < 0.05). Key pathways and genes, including *NPY*, are highlighted.

Upregulated pathways were concentrated in neuropeptide signaling, secretory regulation, and glycosaminoglycan metabolism. GO circular plot analysis (Figure 4A) revealed the enrichment of neuropeptide Y receptor binding (GO:0031841), where *NPY* was the sole enriched gene, alongside positive regulation of epinephrine secretion (GO:0032812), which included VIP (vasoactive intestinal peptide) as a key neuropeptide gene. Additional upregulated GO terms included the regulation of retrograde axon cargo transport (GO:2001017, GO:2001019) (enriched for CALY), peptidoglycan glycosyltransferase activity (GO:0008955), and glucuronylgalactosylproteoglycan 4-beta-N-acetylgalactosaminyltransferase activity (GO:0047237) (both enriched for CSGALNACT1). KEGG Sankey bubble plot analysis (Figure 4B) further corroborated these findings, identifying the activation of the cAMP signaling pathway (hsa04024) and neuroactive ligand–receptor interaction (hsa04080), both of which encompassed *NPY* and VIP as core signaling components. Other upregulated KEGG pathways included glycosaminoglycan biosynthesis of chondroitin sulfate and dermatan sulfate (hsa00532, enriched for CSGALNACT1) and regulation of lipolysis in adipocytes (hsa04923, enriched for *NPY*).

In contrast, downregulated pathways were dominated by mitochondrial energy metabolism and protein homeostasis. GO circular plot analysis (Figure 4A) showed marked suppression of mitochondrial respiratory and energy-generating processes, including the proton-transporting ATP synthase complex (GO:0045263), aerobic respiration (GO:0009060), respirasome (GO:0070469), cytochrome complex (GO:0070069), transporter complex (GO:1990351), and mitochondrial protein-containing complex (GO:0098798), all essential for oxidative phosphorylation. Concurrently, chaperone binding (GO:0051087) and oxidoreduction-driven active transmembrane transporter activity (GO:0015453) were downregulated, indicating compromised protein quality control and metabolic transport machinery. KEGG Sankey bubble plot analysis (Figure 4C) aligned with these GO findings, demonstrating robust suppression of oxidative phosphorylation (hsa00190) and multiple neurodegenerative disease pathways (e.g., prion disease, Parkinson disease, Alzheimer disease, and amyotrophic lateral sclerosis).

These results indicate that, in TLE microglia, upregulated pathways were enriched for neuropeptide- and inflammatory-related functions, whereas downregulated pathways were enriched for oxidative phosphorylation and mitochondrial energy metabolism. The coexistence of these opposing patterns was observed at the transcriptional level; functional interpretation remains limited in the current study. Notably, *NPY* upregulation and enrichment in neuropeptide-related GO terms were shared between microglia and oligodendrocytes. Key inflammation-related DEGs in microglia included immunomodulatory genes (*VIP*, *NPY*, *FKBP5*, and *ZBTB16*), chemokines (*CXCL14*, *CCL8*, and *CCL20*), and immune regulatory factors (*CXCR4*, *FCGR2A*, and *XBP1*), with detailed information listed in Supplementary Table S10.

#### 3.3.2. Oligodendrocytes

Oligodendrocytes in TLE exhibited 14 upregulated and 118 downregulated DEGs. Combined GO and KEGG pathway enrichment analyses were performed, with top enriched terms listed in Supplementary Table S6 (*p* < 0.05).

**Figure 5:**
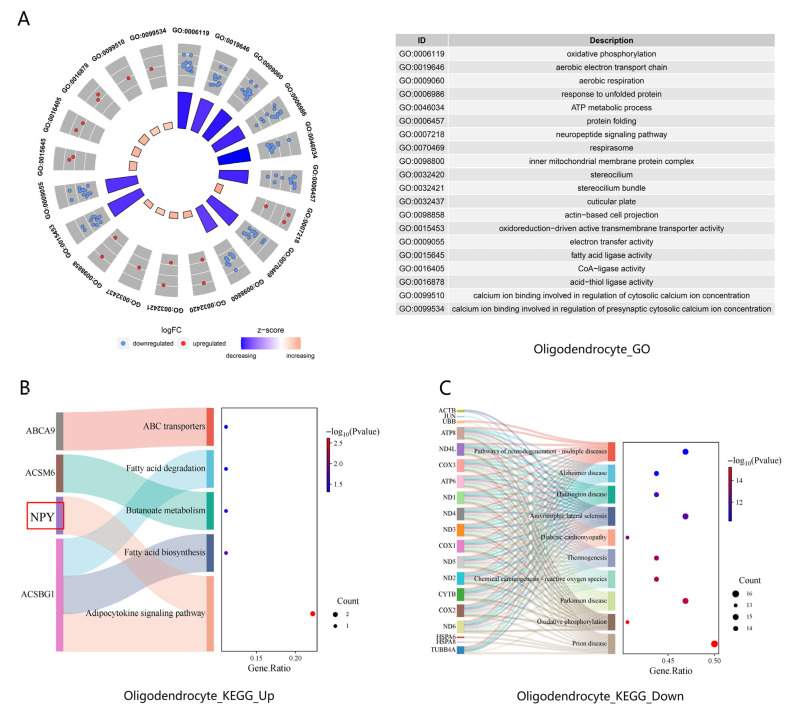
GO and KEGG enrichment analyses of DEGs in oligodendrocytes between TLE and control groups. (**A**) Circular plot showing the top enriched GO terms for upregulated (red) and downregulated (blue) genes in oligodendrocytes (*p* < 0.05). (**B**) Sankey bubble plot of upregulated KEGG pathways (*p* < 0.05). (**C**) Sankey bubble plot of downregulated KEGG pathways (*p* < 0.05). Key pathways and genes, including *NPY*, are highlighted.

Upregulated pathways were concentrated in neuropeptide signaling and lipid metabolism. GO circular plot analysis (Figure 5A) revealed significant enrichment of the neuropeptide signaling pathway (GO:0007218) and neuropeptide Y receptor binding (GO:0031841), both centered on *NPY* alongside PCSK1N and TAC3. Concurrently, lipid metabolic processes were robustly activated, as evidenced by three interconnected GO terms: fatty acid ligase activity (GO:0015645), CoA-ligase activity (GO:0016405), and acid-thiol ligase activity (GO:0016878), all enriched for *ACSBG1* and *ACSM6*. Additional upregulated GO terms included stereocilium (GO:0032420), stereocilium bundle (GO:0032421), and calcium ion binding (GO:0099510). KEGG Sankey bubble plot analysis (Figure 5B) further corroborated these findings, identifying the adipocytokine signaling pathway (hsa04920) as a top upregulated pathway, which included *ACSBG1* and *NPY*, alongside fatty acid biosynthesis (hsa00061), butanoate metabolism (hsa00650), fatty acid degradation (hsa00071), and ABC transporters (hsa02010).

In contrast, downregulated pathways were dominated by mitochondrial energy metabolism and protein homeostasis. GO circular plot analysis (Figure 5A) showed marked suppression of mitochondrial respiratory complexes, including the proton-transporting ATP synthase complex (GO:0045263), aerobic respiration (GO:0009060), respirasome (GO:0070469), and cytochrome complex (GO:0070069), all essential for oxidative phosphorylation. Additionally, protein folding (GO:0006457) and chaperone binding (GO:0051087) were significantly downregulated, with the enrichment of molecular chaperone genes such as *HSP90AA1*, *DNAJA1*, and *BAG3*. KEGG Sankey bubble plot analysis (Figure 5C) aligned with these GO findings, demonstrating robust suppression of oxidative phosphorylation (hsa00190) and multiple neurodegenerative disease pathways (e.g., prion disease, Parkinson disease, Alzheimer disease, and amyotrophic lateral sclerosis).

These results indicate that oligodendrocytes in TLE showed upregulation of lipid-metabolism-related pathways together with downregulation of oxidative phosphorylation- and protein homeostasis-related pathways. The coexistence of these opposing patterns was observed at the transcriptional level; functional interpretation remains limited in the current study. Notably, *NPY* upregulation and enrichment in neuropeptide-related GO terms were observed in both oligodendrocytes and microglia. Selected lipid metabolism-related differentially expressed genes in oligodendrocytes are listed in Supplementary Table S11.

#### 3.3.3. Oligodendrocyte Progenitor Cells

OPCs in TLE exhibited three upregulated and 14 downregulated DEGs. Combined GO and KEGG pathway enrichment analyses were performed, with top enriched terms listed in Supplementary Table S7 (*p* < 0.05).

Upregulated pathways were concentrated in hormone-mediated signaling and stress responses, with *SST* (somatostatin) as the core enriched gene across GO and KEGG analyses (Supplementary Figure S4A,B). Notably, *NPY* was identified as a top upregulated gene in OPCs (log_2_FC = 2.30, adjusted *p* = 0.259) in the differential expression analysis (Supplementary Table S4), though it did not meet the significance threshold and was not enriched in any upregulated pathways.

In contrast, downregulated pathways were dominated by mitochondrial energy metabolism and protein homeostasis, marked by suppression of oxidative phosphorylation and related mitochondrial respiratory complexes, alongside compromised protein quality control (Supplementary Figure S4A,C). As the precursor population of the oligodendrocyte lineage, OPCs shared core mitochondrial energy metabolism impairments with mature oligodendrocytes while activating distinct hormone and stress-related signaling pathways driven primarily by *SST*. The nominal upregulation of *NPY* in OPCs (log_2_FC = 2.30, adjusted *p* = 0.259) did not meet the predefined significance threshold; whether this pattern extends throughout the oligodendrocyte lineage requires investigation in larger cohorts.

#### 3.3.4. Neurons

Neurons in TLE exhibited 236 upregulated and 276 downregulated DEGs. Combined GO and KEGG pathway enrichment analyses were performed, with top enriched terms listed in Supplementary Table S8 (*p* < 0.05).

Upregulated pathways were concentrated in oxidoreductase activity, metabolic processes, and immune regulation, with core genes including *BBOX1*, *HSD11B1*, *FMO5*, and *CPB2* (Supplementary Figure S5A,B). Notably, *NPY* was identified as a top upregulated gene in neurons in the differential expression analysis (Supplementary Table S4, log_2_FC = 1.75, adjusted *p* = 0.0457), though it did not meet our stringent significance threshold and was not enriched in any upregulated pathways.

In contrast, downregulated pathways were dominated by mitochondrial energy metabolism and protein homeostasis, marked by suppression of oxidative phosphorylation and mitochondrial respiratory complexes, alongside compromised protein folding and stress response (Supplementary Figure S5A,C). In neurons, downregulated pathways were enriched for oxidative phosphorylation, mitochondrial respiratory complexes, and protein-folding-related functions, whereas upregulated pathways were associated mainly with oxidoreductase activity, metabolic processes, and immune regulation. *NPY* showed nominal upregulation but did not meet the predefined significance threshold.

#### 3.3.5. Astrocytes

Astrocytes in TLE exhibited zero upregulated and three downregulated DEGs. Due to the extremely low number of astrocytes captured in TLE samples (154 cells, 0.34% of total), reliable differential expression and pathway enrichment analyses were limited. Only three downregulated DEGs were identified, and no significantly upregulated DEGs were detected (Supplementary Table S9). The low abundance of this cell type precluded in-depth functional interpretation, and these findings should be interpreted with caution. Exploratory enrichment analysis identified synaptic-regulation-related terms among the limited astrocyte-associated results (Supplementary Figure S6); however, these findings should be interpreted with caution, given the very low astrocyte abundance in TLE samples.

### 3.4. Cell–Cell Communication Network Alterations

To investigate intercellular communication rewiring in TLE, we performed CellChat analysis on all seven cell types. Global comparison revealed a marked decrease in both the number and strength of intercellular interactions in the TLE samples compared to the controls (Supplementary Figure S7A,B). The total number of inferred interactions dropped from 2119 in controls to 900 in TLE, while the overall interaction strength declined from 87.3 to 38.8 (Supplementary Figure S7A,B), indicating substantial reduction in intercellular communication within the epileptic microenvironment.

**Figure 6:**
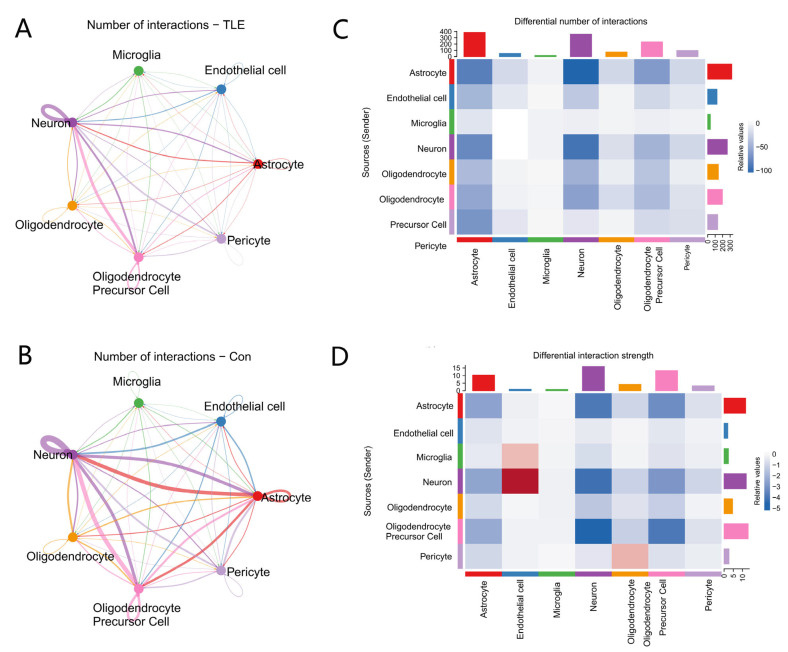
Cell–cell communication network alterations in TLE. (**A**,**B**) Circle plots showing inferred intercellular communication networks in control (**A**) and TLE (**B**) samples. Edge thickness represents interaction strength. The total number of inferred interactions decreased from 2119 in controls to 900 in TLE, with overall interaction strength declining from 87.3 to 38.8 (Supplementary Figure S7). (**C**,**D**) Heatmaps showing the differential number (**C**) and strength (**D**) of interactions between TLE and control groups. Red indicates increased interactions in TLE, and blue indicates decreased interactions. Microglia and oligodendrocytes exhibited relatively low reductions in both interaction number and strength compared to other cell types.

Circle plots comparing control and TLE networks (Figure 6A,B) showed that TLE samples exhibited a markedly sparser network, with general reduction in connectivity across most cell types. Quantitative analysis using differential interaction heatmaps (Figure 6C,D) revealed widespread reductions across multiple cell types. Notably, microglia and oligodendrocytes exhibited relatively low reductions in both interaction number and strength compared to other cell types (Figure 6C), suggesting these glial populations showed relatively smaller reductions in inferred interactions within the suppressed global network.

Microglia and oligodendrocytes showed relatively smaller reductions in inferred interaction number and strength compared to several other cell types (Figure 6C,D). The coexistence of this pattern and glial *NPY* upregulation is noteworthy; however, the present data do not establish that *NPY* directly mediates these inferred communication patterns or that *NPY*^+^ glial subpopulations function as communication hubs. This observation should be considered as hypothesis-generating.

## 4. Discussion

TLE is a complex neurological disorder characterized by multi-cell-type interactions and heterogeneous molecular alterations. In this study, we systematically analyzed the transcriptomic profiles of seven cell types in the temporal cortex of TLE patients. Due to substantial imbalance in neuronal cell numbers between groups and the low abundance of astrocytes and OPCs, we focused our detailed functional analysis on microglia and oligodendrocytes, where microglia counts were relatively comparable and oligodendrocytes remained interpretable despite imbalance. Our findings reveal a striking bidirectional regulation of *NPY*. Specifically, *NPY* was downregulated in most cell types but significantly upregulated in microglia and oligodendrocytes. This identifies *NPY* as a candidate gene of interest associated with glial-associated transcriptional patterns in this TLE dataset.

### 4.1. Glial-Specific Upregulation of NPY in the TLE Cortex

Global transcriptomic analysis revealed that *NPY* was among the top 20 most highly expressed genes in the epileptic brain (Supplementary Figure S1). This is consistent with its well-established role as a key modulator of neuronal excitability and neuroinflammation [17,18,19]. However, our cell-type-specific analysis revealed that this high expression is not solely neuronal; rather, *NPY* was robustly upregulated in microglia and oligodendrocytes under stringent statistical criteria, pointing to previously unrecognized glial sources of *NPY* signaling in the epileptic brain.

Specifically, microglia exhibited a 4.35-fold increase in *NPY* expression (adjusted *p* = 1.12 × 10^−11^), and oligodendrocytes showed a 4.19-fold increase (adjusted *p* = 5.32 × 10^−26^) (Table 3). In contrast, although nominal upregulation was observed in neurons and OPCs, these changes did not meet our stringent significance threshold (Supplementary Table S4). No significant *NPY* expression change was detected in astrocytes, and reliable analysis of endothelial cells and pericytes was precluded by their extremely low abundance in TLE samples (Table 2).

Notably, *NPY* upregulation in microglia and oligodendrocytes was confined to discrete subsets of cells within each population rather than being broadly induced (Supplementary Figure S3), suggesting a cell-state-specific response to the epileptic microenvironment. This pattern is reminiscent of “disease-associated” glial subtypes observed in other neurological disorders [14], where a fraction of glial cells adopt a reactive phenotype characterized by the expression of genes typically associated with neuronal function.

Given the robust statistical support, the relatively comparable microglia counts between groups and the substantially imbalanced but still interpretable oligodendrocyte counts (microglia: 6300 vs. 5151; oligodendrocytes: 20,025 vs. 9729) and the novelty of glial *NPY* upregulation, we focused our subsequent in-depth analysis on these two cell types. Their distinct transcriptional programs and metabolic alterations are discussed in the following sections.

### 4.2. Concurrent Inflammatory Activation and Metabolic Suppression in Microglia

Microglia, identified as one of the two principal sources of *NPY* upregulation in the epileptic brain, exhibited 14 upregulated and 36 downregulated DEGs, with *NPY* ranking among the top upregulated genes (Table 3). Functional enrichment analysis revealed a notable transcriptional pattern: upregulation of neuropeptide signaling and immune pathways occurred alongside downregulation of mitochondrial energy metabolism and protein folding (Figure 4). Thus, microglia in the epileptic cortex displayed concurrent activation of neuropeptide/inflammatory signaling and suppression of oxidative phosphorylation (*p* < 0.05).

This finding adds to recent single-cell studies identifying diverse microglial phenotypes in TLE. He et al. described an MG3 phenotype characterized by iron accumulation and neuroinflammation [14]. In our limited sample, we observed downregulation of oxidative phosphorylation genes accompanying immune-related upregulation, a pattern that has been reported in activated microglia under other neuroinflammatory conditions [22]. However, whether this represents a stable phenotypic shift or a transient adaptive response cannot be determined from cross-sectional transcriptomic data. The functional significance of *NPY* upregulation in microglia remains speculative given our study design. If validated, *NPY* could potentially act in an autocrine or paracrine manner to modulate inflammation, as suggested by in vitro studies showing *NPY* effects on microglial polarization [23,24]. Alternatively, *NPY* upregulation may simply reflect a stress response without functional consequences or could be epiphenomenal to other primary pathological processes.

The enrichment of glycosaminoglycan metabolism pathways in microglia, particularly CSGALNACT1, adds another layer to this complex transcriptional response. Chondroitin sulfate proteoglycans are major components of the perineuronal net and have been implicated in synaptic plasticity and epilepsy [25]. Microglial regulation of extracellular matrix composition may therefore contribute to network hyperexcitability in TLE.

### 4.3. Upregulated Lipid Metabolism with Compromised Energy Production in Oligodendrocytes

Oligodendrocytes, the other major glial population showing robust *NPY* upregulation, displayed 14 upregulated and 118 downregulated DEGs, with *NPY* again ranking among the top upregulated genes (Table 3). Upregulated pathways included lipid metabolism and neuroactive ligand–receptor interactions, while downregulated pathways encompassed oxidative phosphorylation and protein processing (Figure 5). These results indicate that oligodendrocytes in TLE showed upregulation of lipid metabolism-related pathways together with downregulation of oxidative phosphorylation-related pathways. The coexistence of these opposing patterns was observed at the transcriptional level; whether this reflects metabolic tension or functional consequences for myelination requires experimental validation [26,27].

Our observations are consistent with emerging evidence of white matter pathology in TLE, including DTI-identified microstructural abnormalities [26,27] and oligodendrocyte lineage vulnerability in epileptogenic tissue [28]. The co-occurrence of *NPY* upregulation with metabolic pathway alterations may suggest a stress-induced transcriptional response, though whether this is adaptive or maladaptive requires experimental investigation. Although *NPY* upregulation in OPCs did not reach statistical significance under our stringent criteria, the nominal upregulation observed (log_2_FC = 4.22) suggests that this stress response may potentially extend throughout the oligodendrocyte lineage, warranting investigation in larger cohorts.

### 4.4. Global Suppression of Intercellular Communication with Relative Preservation of Glial Connectivity

The CellChat analysis revealed a marked global decrease in intercellular communication in TLE, with total inferred interactions reduced by 57.5% (from 2119 in controls to 900 in TLE) and overall interaction strength declining by 55.6% (from 87.3 to 38.8) (Supplementary Figure S7). This widespread suppression challenges the conventional assumption that epilepsy solely involves hyperexcitable network enhancement and suggests that chronic pathology may lead to extensive disruption of both synaptic and non-synaptic cell–cell signaling.

Despite this global suppression, microglia and oligodendrocytes exhibited relatively low reductions in both interaction number and strength compared to other cell types (Figure 6C, D). When coupled with the selective upregulation of *NPY* in discrete subsets of these glial populations, this relative preservation of inferred glial connectivity, observed alongside glial NPY upregulation, generates a testable hypothesis for future studies. However, the present data do not establish a functional role for *NPY* in maintaining connectivity, nor do they identify the specific ligand–receptor interactions underlying these patterns. Given that *NPY* expression was confined to a subset of cells rather than broadly induced, these *NPY*^+^ glial cells may mark a specific transcriptional state; whether this state corresponds to specialized functional properties requires experimental validation.

Several potential mechanisms could underlie this resilience. First, these cells might sustain local metabolic support through lactate shuttling or other metabolite exchange, which is critical for meeting the heightened energy demands of active neurons in the epileptic microenvironment [29,30]. Second, *NPY* itself has been shown to suppress pro-inflammatory cytokine production and modulate glial activation states [23,24], suggesting that *NPY*^+^ glia could contribute to inflammation containment. Third, these cells may participate in myelin integrity surveillance and repair, a function particularly relevant given the metabolic tension we observed in oligodendrocytes. However, the specific ligand–receptor interactions mediating this relative preservation remain to be elucidated. Future studies using spatial transcriptomics, ligand–receptor perturbation experiments, or cell-type-specific knockout models are needed to determine whether *NPY* directly contributes to maintaining glial connectivity or merely marks a broader stress-resistant cell state.

### 4.5. Glial NPY Upregulation: A Marker of Cell-State-Specific Response

The observation that *NPY* upregulation was confined to a subset of microglia and oligodendrocytes rather than being broadly induced across all cells of these types is a key finding. This pattern suggests that *NPY* expression marks a specific glial cell state induced by the epileptic microenvironment rather than representing a general transcriptional response. Such cell-state-specific markers have been described in other neurological disorders, where they often identify disease-associated glial phenotypes with distinct functional properties [31,32,33,34].

The functional implications of glial *NPY* expression remain to be elucidated. *NPY* could act in an autocrine manner to modulate glial stress responses or in a paracrine manner to influence neighboring neurons or glia. The relative preservation of microglial and oligodendroglial inferred connectivity, observed alongside glial *NPY* upregulation, generates a testable hypothesis for future studies. However, the present data do not establish that *NPY*-expressing cells serve as local communication hubs. Future studies using spatial transcriptomics or cell-type-specific knockout models are needed to dissect the precise role of microglial and oligodendroglial *NPY* in epilepsy.

### 4.6. Limitations

Several limitations warrant acknowledgment. First, our sample size is relatively small, with only three TLE patients and two controls. This may limit the generalizability of our findings and necessitates validation in larger independent cohorts. Second, we analyzed only temporal cortex tissues. The hippocampus, which is a key epileptogenic zone in TLE, was not included in this study, and molecular alterations in the hippocampus may differ from those in the adjacent cortex. Third, the substantial imbalance in neuronal cell numbers between TLE and control groups (14,923 vs. 2305 cells) precluded reliable differential expression analysis in this critical cell type. Neuronal DEGs are provided in Supplementary Table S4, but these results should be interpreted with caution. Fourth, the low abundance of astrocytes limited our ability to analyze this cell type in depth. Astrocytes constituted only 0.34% of cells in TLE samples versus 4.26% in controls. Similarly, the low number of OPCs in controls (1115 cells) and the imbalance in OPC numbers (3594 in TLE vs. 1115 in controls) restricted our analysis. Fifth, while we identified *NPY* as a candidate gene of interest and assessed its upregulation by qPCR, protein-level validation in specific cell types would strengthen the findings. Techniques such as immunohistochemistry or RNAscope could provide this validation in future studies. Sixth, we acknowledge that antiepileptic medications may influence gene expression profiles; however, all TLE patients underwent preoperative AED withdrawal (3–7 days) for video EEG monitoring, minimizing acute drug effects on tissue transcriptomes [20,21]. The observed glial-specific NPY upregulation likely reflects disease-associated changes rather than drug effects, particularly given that control subjects were AED-naïve. Future studies with AED-matched controls are needed to fully disentangle these effects. Seventh, and fundamentally, our study is descriptive and correlative. While we identify compelling molecular signatures including concurrent activation of inflammatory signaling and suppression of oxidative metabolism in microglia, as well as upregulated lipid metabolism with compromised energy production in oligodendrocytes, these hypotheses regarding functional roles are based on transcriptional inferences. A summary of cell-type-specific metabolic pathway alterations is provided in Supplementary Table S12. The causal relationships and exact mechanisms remain to be tested experimentally. Future studies employing cell-type-specific knockout models, pharmacological interventions, or functional assays are needed to verify the precise role of glial *NPY* signaling and the metabolic alterations that we describe in TLE pathogenesis.

## 5. Conclusions

In summary, this exploratory snRNA-seq study suggests that *NPY* upregulation in the TLE temporal cortex is the most evident in microglia and oligodendrocytes under the analytical criteria used here. These glial populations also showed distinct transcriptomic patterns: microglia exhibited enrichment of neuropeptide- and inflammatory-related pathways, together with reduced oxidative phosphorylation signatures, whereas oligodendrocytes exhibited altered lipid metabolism, together with reduced mitochondrial energy-related signatures. Inferred intercellular communication was globally reduced in the TLE samples. Given the limited sample size, cell-count imbalance, and a lack of functional validation, these observations should be interpreted as preliminary and hypothesis-generating. Nevertheless, they provide a cell-type-resolved descriptive framework that may inform future mechanistic and validation studies of glial-associated responses in human epilepsy.

## Figures and Tables

**Table 1:** Single-nucleus sequencing quality control metrics.

Sample ID	Total Reads (×10^6^)	Mapping Rate (%)	Cells After Filtering	Median UMI/Cell (IQR)	Median Genes/Cell (IQR)	Median Mitochondrial Ratio (%, IQR)
TLE_01	428.6	83.5	14,236	2089	1298	4.2
TLE_02	456.2	85.1	15,128	2215	1356	3.8
TLE_03	432.9	84.3	14,872	2156	1332	4.5
Con_01	418.5	82.8	12,345	2103	1289	3.5
Con_02	420.6	83.7	12,351	2198	1368	3.2

Abbreviations: TLE, temporal lobe epilepsy; UMI, unique molecular identifier; IQR, interquartile range. TLE_01–TLE_03: TLE patient samples; Con_01–Con_02: non-epileptic control samples. Sample-level metrics are raw values; cell-level metrics are median (IQR). Nuclei were filtered using criteria described in Section 2.

**Table 2:** Cell type counts and proportions in TLE and control groups.

Cell Type	TLE (*n* = 3)	TLE (%)	Control (*n* = 2)	Control (%)
Neuron	14,923	33.09	2305	10.56
Astrocyte	154	0.34	930	4.26
Microglia	6300	13.97	5151	23.60
Oligodendrocyte	20,025	44.40	9729	44.57
OPC	3594	7.97	1115	5.11
Endothelial cell	48	0.11	1767	8.10
Pericyte	60	0.13	831	3.81
Total	45,104	100.00	21,828	100.00

**Table 3:** List of the top five upregulated and downregulated genes in microglia and oligodendrocyte clusters.

Microglia		Oligodendrocyte	
Gene Name	Log FC	Gene Name	Log FC
*VIP*	4.61	*TAC3*	4.33
*NPY*	4.36	*NPY*	4.19
*DLEU7*	4.23	*CALB2*	2.97
*CALY*	3.38	*MDFIC2*	2.86
*CXCL14*	2.80	*CXCL14*	2.73
*BAG3*	−7.30	*HSPA6*	−7.63
*HSPH1*	−6.55	*HSPB1*	−6.22
*ZFAND2A*	−6.40	*BAG3*	−6.04
*HSPD1*	−6.21	*TM4SF1*	−5.96
*MT-ATP8*	−6.08	*MT-ATP8*	−5.59

## Data Availability

The data presented in this study are available on request from the corresponding author due to privacy/ethical restrictions.

## Supplementary Materials

The following supporting information can be downloaded at: https://www.mdpi.com/article/10.3390/biology15080627/s1, Figure S1. Violin plots of the top 20 differentially expressed genes in bulk temporal cortex tissues from TLE patients and non-epileptic controls. Figure S2. Heatmap showing the top five most highly expressed genes in each major cell type. Figure S3. t-SNE visualization of cell type distribution and NPY expression in TLE and control groups. Figure S4. GO and KEGG enrichment analyses of DEGs in OPCs between TLE and control groups. Figure S5. GO and KEGG enrichment analyses of DEGs in neurons between TLE and control groups. Figure S6. GO and KEGG enrichment analyses of DEGs in astrocytes between TLE and control groups. Figure S7. Global reduction of intercellular communication between TLE and control groups. Table S1. Inclusion and exclusion criteria for TLE patients and control subjects. Table S2. Clinical and pathological characteristics of all subjects. Table S3. Primer sequences used for qPCR validation. Table S4. Cell-type-specific differential expression of NPY across all cell types. Table S5. Complete list of enriched GO terms and KEGG pathways in microglia. Table S6. Complete list of enriched GO terms and KEGG pathways in oligodendrocytes. Table S7. Complete list of enriched GO terms and KEGG pathways in OPCs. Table S8. Complete list of enriched GO terms and KEGG pathways in neurons. Table S9. Complete list of enriched GO terms and KEGG pathways in astrocytes. Table S10. Selected inflammation-related differentially expressed genes in microglia. Table S11. Selected lipid metabolism-related differentially expressed genes in oligodendrocytes. Table S12. Summary of cell-type-specific metabolic pathway alterations in TLE.
