# Supplementary material for: Exploratory Single-Nucleus RNA Sequencing Suggests Glial-Specific NPY Upregulation and Cell-Type-Specific Metabolic Alterations in Temporal Lobe Epilepsy
Source: Biology (Basel). 2026 Apr 16;15(8):627. doi: 10.3390/biology15080627 (PMC13114130; doi:10.3390/biology15080627)
Supplement: Supplementary file 1 [file biology-15-00627-s001.zip › Supplementary Table S4. Cell-type-specific differential expression of NPY across all cell types..pdf]

Supplementary Table S4. Cell-type-specific differential expression of NPY across all cell types.

Cell Type	log_2FC	Adjusted P	Significance
Microglia	**4.35**	$1.12×10^{-11}$	Yes
Oligodendrocyte	4.19	$5.32×10^{-26}$	Yes
Neuron	1.75	0.0457	No
OPC	4.22	0.259	No
Astrocyte	-	-	No
Endothelial	-	-	No (low counts)
Pericyte	-	-	No (low counts)

Includes log_2FC and adjusted P values for NPY in each cell type. Nominal changes not meeting the significance threshold ($|log_2FC| > 1$, adjusted $P < 0.01$) are indicated. For endothelial cells and pericytes, reliable analysis was precluded by low cell counts.
