# Supplementary material for: Exploratory Single-Nucleus RNA Sequencing Suggests Glial-Specific NPY Upregulation and Cell-Type-Specific Metabolic Alterations in Temporal Lobe Epilepsy
Source: Biology (Basel). 2026 Apr 16;15(8):627. doi: 10.3390/biology15080627 (PMC13114130; doi:10.3390/biology15080627)
Supplement: Supplementary file 1 [file biology-15-00627-s001.zip › Supplementary Table S10. Selected inflammation-related differentially expressed genes in microglia.pdf]

Supplementary Table S10. Selected inflammation-related differentially expressed genes in microglia

Gene	Cell Type	adj.*P*	Inflammation-Related Function / Pathway
**Upregulated**			
VIP	Microglia	0.00136	Vasoactive intestinal peptide; immunomodulatory, anti-inflammatory
NPY	Microglia	1.12×10^{-11}	Neuropeptide Y; modulates microglial activation and cytokine production
CXCL14	Microglia	0.00972	Chemokine; involved in immune cell recruitment and inflammation
FKBP5	Microglia	2.21×10^{-17}	Immunophilin; regulates glucocorticoid receptor sensitivity and inflammatory responses
ZBTB16	Microglia	1.51×10^{-11}	Zinc finger transcription factor; implicated in immune regulation and inflammation
**Downregulated**			
CXCR4	Microglia	0.00845	Chemokine receptor; mediates microglial chemotaxis and neuroinflammation
CCL8	Microglia	0.01026	Chemokine; monocyte chemoattractant, involved in inflammatory cell recruitment
CCL20	Microglia	1.00×10^{-4}	Chemokine; attracts lymphocytes and dendritic cells, associated with neuroinflammation
FCGR2A	Microglia	0.00391	Fc gamma receptor; mediates antibody-dependent immune responses and phagocytosis
XBP1	Microglia	0.01749	Transcription factor; regulates unfolded protein response and inflammatory signaling

Footnote: Genes were selected based on literature review of inflammatory pathways. DEGs meeting the predefined significance threshold ($|\log_2FC| > 1$, adj.$P < 0.01$) are included; CCL8 (adj.$P = 0.01026$) is retained as a representative inflammatory chemokine.
