# Supplementary material for: Exploratory Single-Nucleus RNA Sequencing Suggests Glial-Specific NPY Upregulation and Cell-Type-Specific Metabolic Alterations in Temporal Lobe Epilepsy
Source: Biology (Basel). 2026 Apr 16;15(8):627. doi: 10.3390/biology15080627 (PMC13114130; doi:10.3390/biology15080627)
Supplement: Supplementary file 1 [file biology-15-00627-s001.zip › Supplementary Table S1. Inclusion and exclusion criteria for TLE patients and control subjects..pdf]

Supplementary Table S1. Inclusion and exclusion criteria for TLE patients and control subjects.

Group	Inclusion Criteria	Exclusion Criteria
TLE patients （n=3）	1. Age 18–65 years<br>2. Diagnosed with drug-resistant epilepsy according to ILAE criteria<br>3. Montreal Cognitive Assessment (MoCA) score ≥ 26<br>4. Epileptogenic zone localized by at least two of the following: long-term video EEG, electrocorticography (ECoG), depth EEG, CT, MRI (plain/enhanced/functional), PET-CT<br>5. Failed to achieve seizure control after ≥2 years of adequate treatment with ≥2 first-line anti-seizure medications<br>6. Pathologically confirmed hippocampal sclerosis post-surgery	1. History of cardiac, pulmonary, hepatic, renal, or other major organ dysfunction
Control subjects （n=2）	1. Age 18–65 years<br>2. No history of seizures or epilepsy<br>3. No family history of epilepsy<br>4. No contraindications for MRI<br>5. Montreal Cognitive Assessment (MoCA) score ≥ 26<br>6. No evidence of dementia<br>7. No history of cardiac, pulmonary, hepatic, renal, or other major organ dysfunction<br>8. For tumor patients: Postoperative pathology confirmed benign lesion (meningioma, WHO grade I) with no tumor cell infiltration in collected tissue<br>9. For trauma patients: No diffuse axonal injury or severe brainstem injury	– Tissue samples collected >2 cm from primary injury site (trauma) or >2 cm from tumor margin (tumor)

Includes clinical, radiological, and histopathological criteria for TLE patients and controls. See the Methods section for details.
