# Supplementary material for: Exploratory Single-Nucleus RNA Sequencing Suggests Glial-Specific NPY Upregulation and Cell-Type-Specific Metabolic Alterations in Temporal Lobe Epilepsy
Source: Biology (Basel). 2026 Apr 16;15(8):627. doi: 10.3390/biology15080627 (PMC13114130; doi:10.3390/biology15080627)
Supplement: Supplementary file 1 [file biology-15-00627-s001.zip › Supplementary Table S2. Clinical and pathological characteristics of all subjects..pdf]

Supplementary Table S2. Clinical and pathological characteristics of all subjects

Group	Age (years)	Sex	Disease duration (years)	Seizure type	MoCA score	Preoperative EEG	MRI/PET findings	Pathological diagnosis	Tissue source	Antiepileptic medication(s)
TLE	26	F	16	Focal mesial temporal lobe seizures	27	Epileptiform discharges	Localized to mesial temporal lobe	Temporal lobe epilepsy with hippocampal sclerosis	Epilepsy surgery	Levetiracetam 0.5g BID; Lamotrigine 50mg BID; Oxcarbazepine 0.45g BID
TLE	36	F	22	Focal mesial temporal lobe seizures	28	Epileptiform discharges	Localized to mesial temporal lobe	Temporal lobe epilepsy with hippocampal sclerosis	Epilepsy surgery	Lamotrigine 100mg BID; Levetiracetam 0.5g BID; Oxcarbazepine 0.3g BID; Sodium valproate 0.25g BID
TLE	34	M	12	Focal mesial temporal lobe seizures	26	Epileptiform discharges	Localized to mesial temporal lobe	Temporal lobe epilepsy with hippocampal sclerosis	Epilepsy surgery	Oxcarbazepine 0.6g (morning) / 0.75g (evening); Levetiracetam 1g (morning) / 1.25g (evening); Perampanel 2mg (evening)
Control	18	M	-	-	27	No epileptiform discharges	Normal (adjacent to meningioma, >2 cm from tumor margin)	Meningioma (WHO grade I), no tumor infiltration in collected tissue	Meningioma surgery	None
Control	50	F	-	-	27	No epileptiform discharges	Normal (adjacent to contused area, >2 cm from injury site)	No pathological abnormalities; no evidence of diffuse axonal injury	Decompressive craniectomy for traumatic brain injury (fall)	None

Abbreviations: TLE, temporal lobe epilepsy; M, male; F, female; MoCA, Montreal Cognitive Assessment; EEG, electroencephalography; MRI, magnetic resonance imaging;

PET, positron emission tomography; WHO, World Health Organization; TBI, traumatic brain injury.

All TLE patients underwent preoperative antiepileptic drug (AED) tapering or withdrawal 3-7 days prior to surgery for video-EEG monitoring, following standard clinical protocols (20,21). This preoperative withdrawal period minimized acute drug effects on brain tissue gene expression.

AED doses are reported as prescribed daily dose at time of admission. BID, twice daily; TID, three times daily. Doses with morning/evening differences are indicated as morning dose/evening dose.

Control subjects were not receiving any chronic pharmacological treatment at the time of surgery.
