# Supplementary material for: Exploratory Single-Nucleus RNA Sequencing Suggests Glial-Specific NPY Upregulation and Cell-Type-Specific Metabolic Alterations in Temporal Lobe Epilepsy
Source: Biology (Basel). 2026 Apr 16;15(8):627. doi: 10.3390/biology15080627 (PMC13114130; doi:10.3390/biology15080627)
Supplement: Supplementary file 1 [file biology-15-00627-s001.zip › Supplementary Table S3. Primer sequences used for qPCR validation..pdf]

Supplementary Table S3. Primer sequences used for qPCR validation.

Gene	Forward Primer (5'→3')	Reverse Primer (5'→3')	Amplicon size (bp)	Tm (°C)	Efficiency (%)
NPY	GGAAAACGATCCAGCCC	AGACAGGGTCTTCAAGCCGAGTT	91	60.04 (F), 59.96 (R)	95–105
HPRT1	TGGATATAAGCCAGACTTTGTT	GGAGCGATGTCAATAGGACTCCAG	193	60.05 (F), 58.25 (R)	95–105

Primers were designed using Primer-BLAST (NCBI). HPRT1 was used as an endogenous reference gene. Melting temperatures (Tm) are indicated for forward (F) and reverse (R) primers separately. All primer pairs exhibited amplification efficiencies within the acceptable range of 95–105%.
