# Supplementary material for: Exploratory Single-Nucleus RNA Sequencing Suggests Glial-Specific NPY Upregulation and Cell-Type-Specific Metabolic Alterations in Temporal Lobe Epilepsy
Source: Biology (Basel). 2026 Apr 16;15(8):627. doi: 10.3390/biology15080627 (PMC13114130; doi:10.3390/biology15080627)
Supplement: Supplementary file 1 [file biology-15-00627-s001.zip › Supplementary Table S11. Selected lipid metabolism-related differentially expressed genes in oligodendrocytes.pdf]

Supplementary Table S11. Selected lipid metabolism-related differentially expressed genes in oligodendrocytes

Gene	Cell Type	$\log_2$FC	adj.*P*	Lipid Metabolism Pathway / Function
**Upregulated**				
ACSM6	Oligodendrocytes	2.26	4.71×10^{-9}	Fatty acid activation / acyl-CoA synthesis
ACSBG1	Oligodendrocytes	2.09	8.25×10^{-4}	Long-chain fatty acid metabolism
ABCA9	Oligodendrocytes	2.06	4.84×10^{-2}	Lipid transport, cholesterol efflux
**Downregulated**				
SCD	Oligodendrocytes	-2.28	1.36×10^{-9}	Fatty acid desaturation
PLP1	Oligodendrocytes	-1.91	2.52×10^{-7}	Myelin lipid composition
DBI	Oligodendrocytes	-2.15	2.52×10^{-10}	Acyl-CoA binding, lipid metabolism

Footnote: Genes were selected based on GO/KEGG enrichment analysis and literature review. Only DEGs meeting the significance threshold ($|\log_2 FC| > 1$, adj.$P < 0.01$) are included, except for ABCA9 (adj.$P = 0.048$) which is included as a representative lipid transport-related gene.
