# Supplementary material for: Exploratory Single-Nucleus RNA Sequencing Suggests Glial-Specific NPY Upregulation and Cell-Type-Specific Metabolic Alterations in Temporal Lobe Epilepsy
Source: Biology (Basel). 2026 Apr 16;15(8):627. doi: 10.3390/biology15080627 (PMC13114130; doi:10.3390/biology15080627)
Supplement: Supplementary file 1 [file biology-15-00627-s001.zip › Supplementary Table S12.Summary of cell-type-specific metabolic pathway alterations in TLE.pdf]

**Supplementary Table S12. Summary of cell-type-specific metabolic pathway alterations in TLE**

| Cell Type | Upregulated Metabolic Pathways / Signatures | Downregulated Metabolic Pathways / Signatures |
|---|---|---|
| Microglia | Neuropeptide signaling (NPY, VIP); Glycosaminoglycan metabolism (CSGALNACT1); cAMP signaling pathway; Neuroactive ligand - receptor interaction | Oxidative phosphorylation; Mitochondrial respiratory complexes; Protein folding / chaperone binding; Cytochrome complex |
| Oligodendrocytes | Neuropeptide signaling (NPY, TAC3); Lipid metabolism (fatty acid ligase, CoA - ligase); Adipocytokine signaling; Fatty acid biosynthesis / degradation; ABC transporters | Oxidative phosphorylation; Mitochondrial respiratory complexes; Protein folding / chaperone binding; Cytochrome complex; Transporter complex |
| OPCs | Hormone - mediated signaling (SST); Response to stress / pH / hyperosmotic; Hormone activity | Oxidative phosphorylation; Mitochondrial respiratory chain complex I Cytochrome complex; Transporter complex; Response to unfolded protein |
| Neurons | Oxidoreductase activity (FMO5, HSD11B1, BBOX1); NADP binding; Complement and coagulation cascades (CPB2, C4BPA); Ferroptosis (CP) | Oxidative phosphorylation; Mitochondrial protein complex; Respirasome; Protein folding / chaperone binding; Response to oxidative stress |
| Astrocytes | Retrograde endocannabinoid signaling (CNR1); Rap1 signaling pathway; Thermogenesis; Neuroactive ligand - receptor interaction; Positive regulation of presynaptic signaling | (Limited analysis due to very low cell abundance; only few DEGs identified) |

Footnote: Pathways were derived from GO/KEGG enrichment analyses of differentially expressed genes in each cell type (nominal $P$ <0.05). Oxidative phosphorylation and mitochondrial energy metabolism were consistently downregulated across microglia, oligodendrocytes, OPCs, and neurons, representing a shared metabolic vulnerability signature. Microglia and oligodendrocytes showed distinct upregulated pathways related to neuropeptide signaling and lipid metabolism, respectively. Astrocyte analysis was constrained by the low number of cells captured in TLE samples (154 cells, 0.34% of total); therefore, these findings should be interpreted with caution. All transcriptomic patterns are candidate signatures of metabolic vulnerability and require functional validation.
