# Supplementary figures and images for: Exploratory Single-Nucleus RNA Sequencing Suggests Glial-Specific NPY Upregulation and Cell-Type-Specific Metabolic Alterations in Temporal Lobe Epilepsy
Source: Biology (Basel). 2026 Apr 16;15(8):627. doi: 10.3390/biology15080627 (PMC13114130; doi:10.3390/biology15080627)

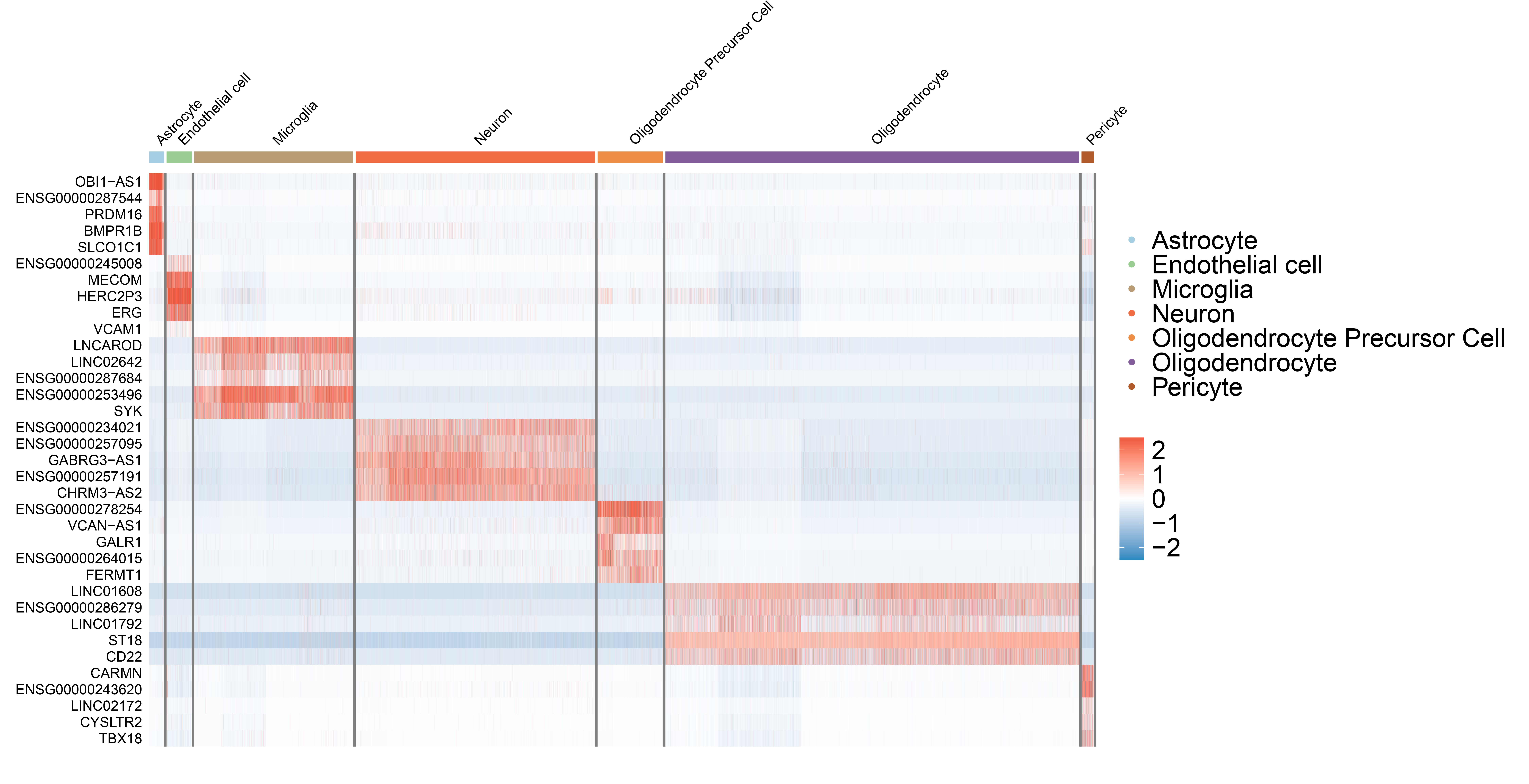

Supplement: Supplementary file 1 [file biology-15-00627-s001.zip › Supplementary Figure S2. Heatmap showing the top five most highly expressed genes in each major cell type.jpg]

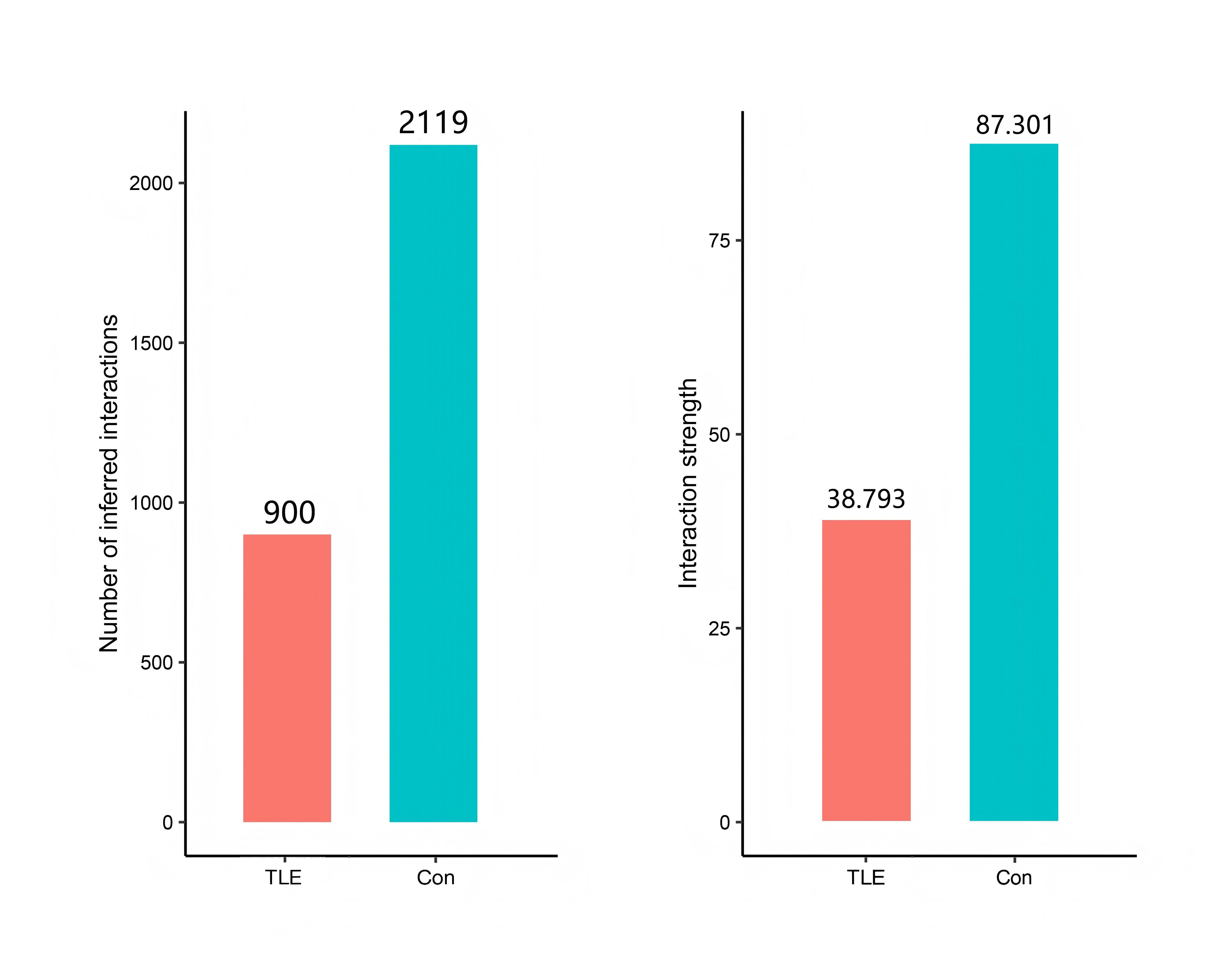

Supplement: Supplementary file 1 [file biology-15-00627-s001.zip › Supplementary Figure S7. Global reduction of intercellular communication between TLE and control groups.jpg]
